# Supplementary material for: Materials and Spin Characteristics of Nanodiamonds Partially Covered with Amino Groups and Embedded with Nitrogen-Vacancy Color Centers
Source: ACS Omega. 2026 Aug 24;11(35):53440–51. doi: 10.1021/acsomega.6c06903 (PMC13563617; doi:10.1021/acsomega.6c06903)
Supplement: Supplementary file 1 [file ao6c06903_si_001.pdf]

Supporting Information: Materials and spin characteristics of nanodiamonds partially covered by amino groups and embedded with nitrogen-vacancy color centers

Nikoletta Jegenyes,[†] Vladimir Verkhovlyuk,[†] Szabolcs Czene,[†,‡] Attila Csáki,[†] Sándor Kollarics,[†,¶,§] Olga Krafcsik,[∥,⊥] Zsolt Czigány,[⊥] David Beke,[*,†,#] and Adam Gali[*,†,∥,@]

†*HUN-REN Wigner Research Centre for Physics, P.O. Box 49, H-1525 Budapest, Hungary*
‡*Doctoral School on Materials Sciences and Technologies, Óbuda University, Bécsi út 96/b, H-1034 Budapest, Hungary*
¶*SOLEIL Synchrotron, L'Orme des Merisiers, RD128, Saint Aubin, France, 91190*
§*Department of Physics, Institute of Physics, Budapest University of Technology and Economics, Műegyetem rakpart 3., H-1111 Budapest, Hungary*
∥*Department of Atomic Physics, Institute of Physics, Budapest University of Technology and Economics, Műegyetem rakpart 3., H-1111 Budapest, Hungary*
⊥*Institute of Technical Physics and Materials Science, HUN-REN Centre for Energy Research, P.O. Box 49, H-1525 Budapest, Hungary*
#*Kandó Kálmán Faculty of Electrical Engineering, Óbuda University, Tavaszmező u. 17., H-1084 Budapest, Hungary*
@*MTA–WFK Lendület "Momentum" Semiconductor Nanostructures Research Group, P.O. Box 49, H-1525 Budapest, Hungary*

E-mail: beke.david@wigner.hun-ren.hu; gali.adam@wigner.hun-ren.hu

Abstract

This document contains supporting morphological, structural, vibrational, X-ray photoelectron, and photoluminescence data for the main text.

1 Analysis by SEM, HRTEM, and EDS

The SEM images clearly show the non-spherical shape of the FNDs in Fig. S1. Fig. S2 presents the EDS spectra of ND10 as-received and washed samples as obtained by SEM.

The presence of sulfur is evident in the as-received samples. This observation was also proven by the EDS analysis performed in TEM (Fig. S3).

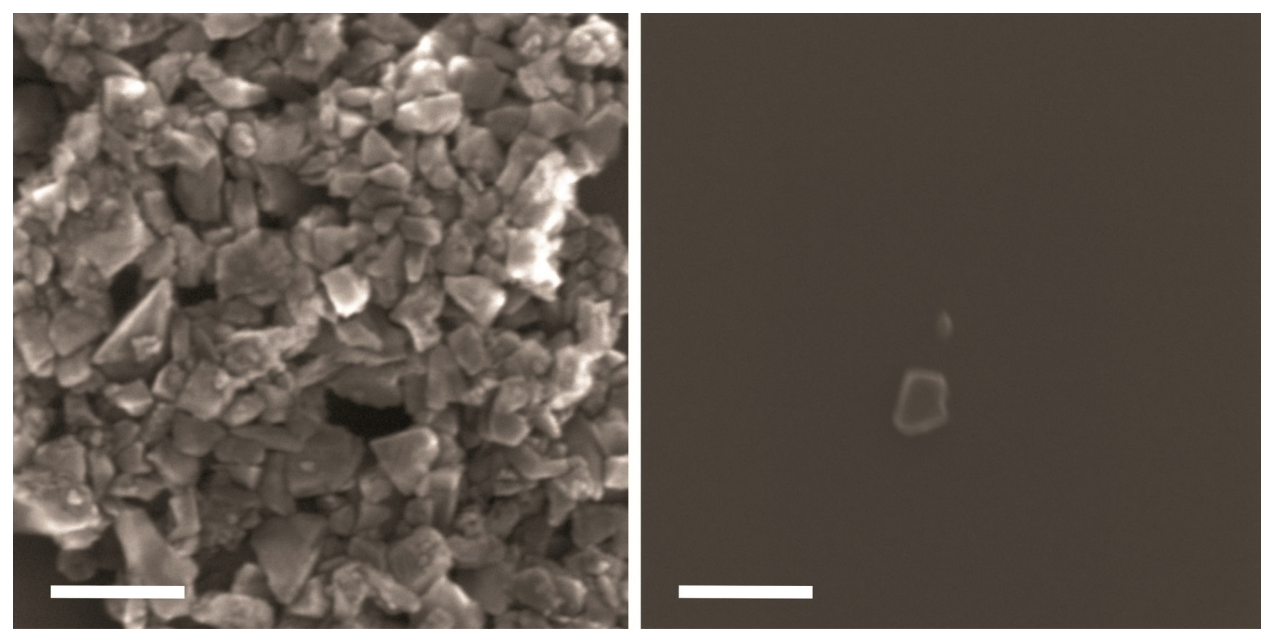

Figure S1: SEM image of ND90 ensemble and individual particles. The white line corresponds to 200 nm.

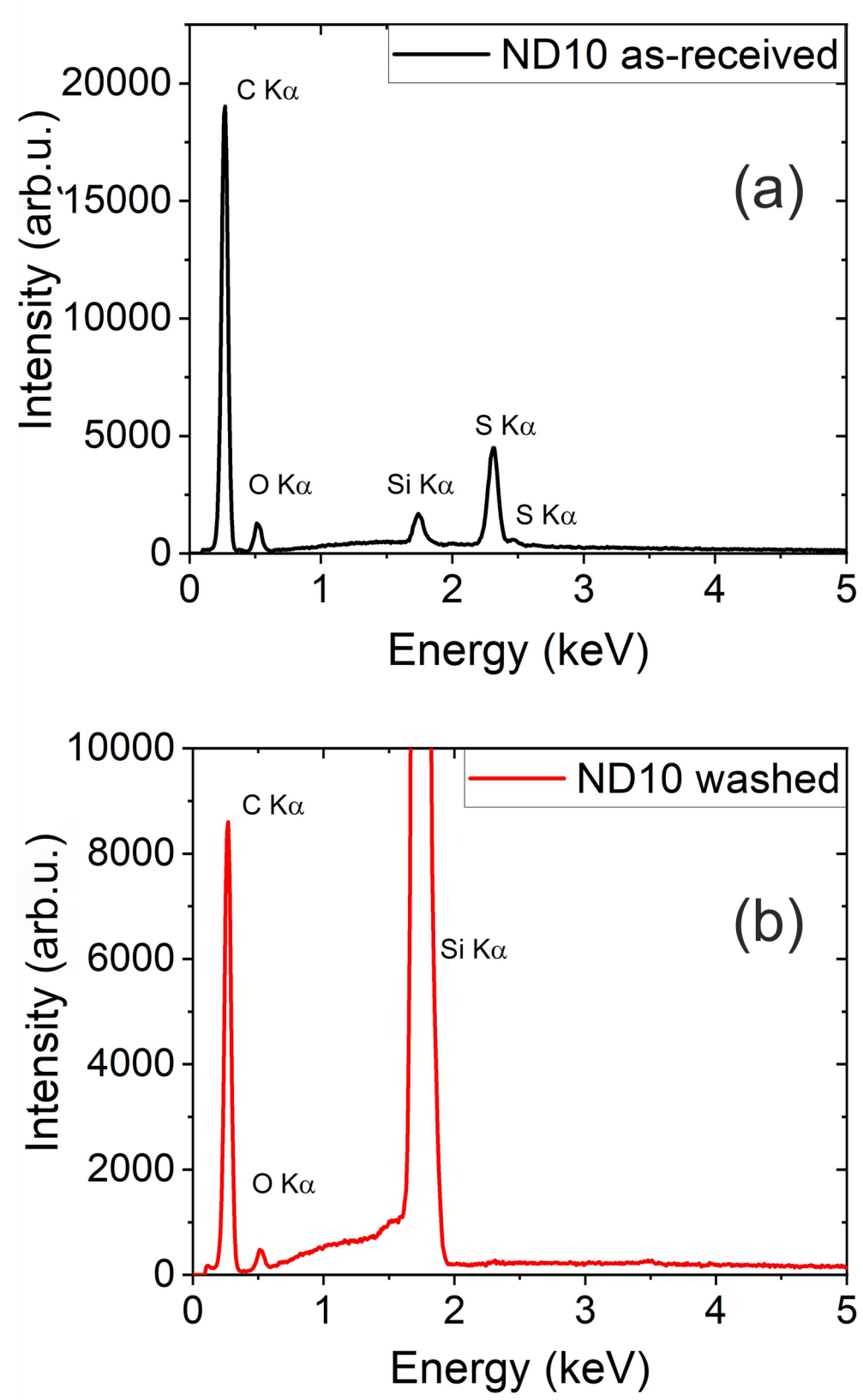

Figure S2: EDS spectra of (a) ND10 as-received and (b) ND10 washed particles.

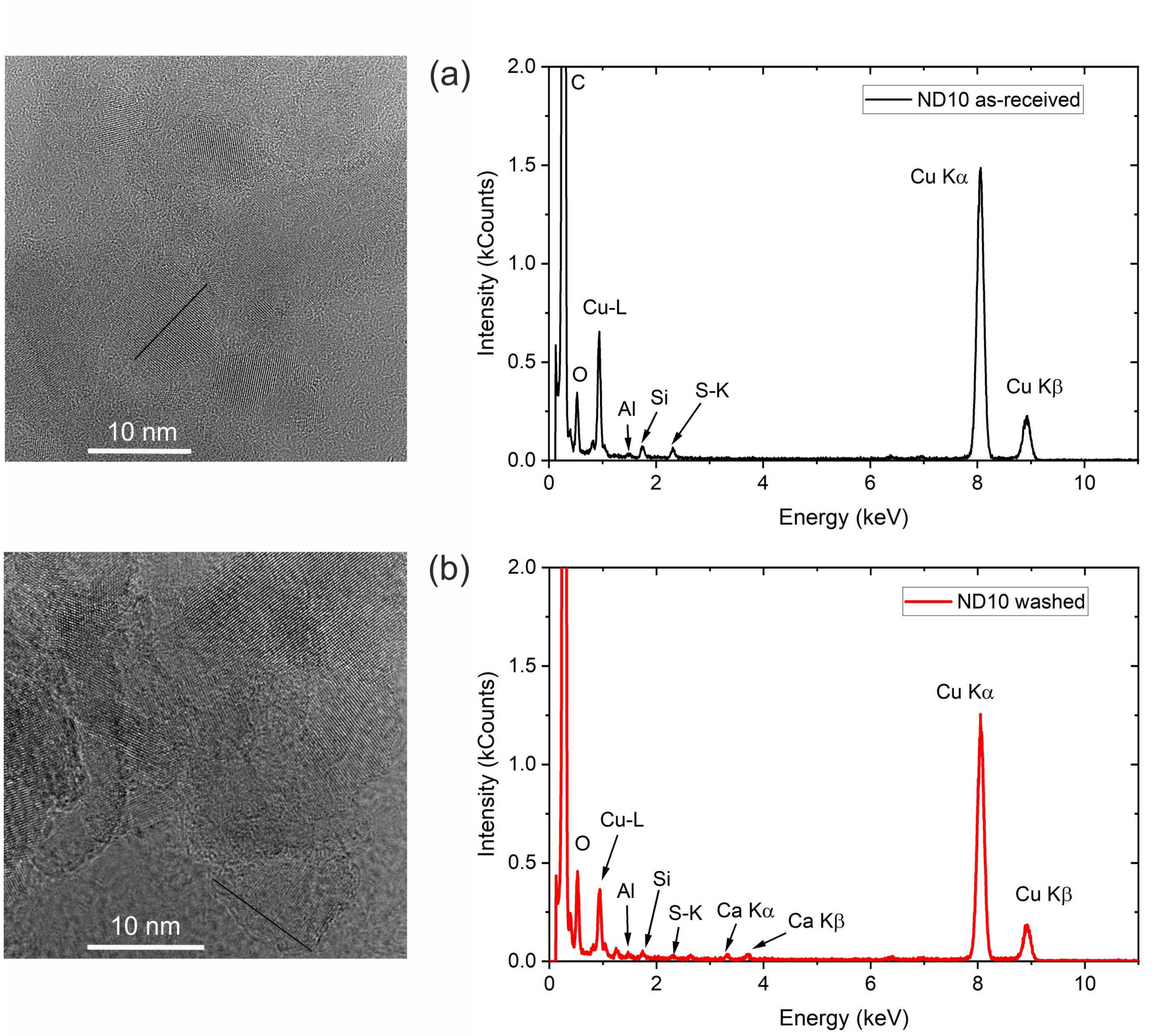

Figure S3: HRTEM image and TEM-EDS analysis of (a) ND10 as-received and (b) ND10 washed particles. The scale corresponds to 10 nm. The black line indicates the measurement of the size of the nanodiamond particle.

2 FTIR supporting data

In Tables S1–S7, the vibrational bands identified in the FTIR spectra (shown in Figure 2 of the main text and Figure S4) are summarized together with their corresponding surface functional group assignments, based on standard FTIR band assignments. FTIR measurements were performed on as-received, washed, hydroxyl-terminated, and amino-terminated samples of the ND10, ND50, and ND90 FNDs. For the ND30, ND40, ND70, and ND140 FNDs, the washed samples were not examined. The FTIR spectra of ND40 closely resembled those of ND30; therefore, the ND40 spectra are not presented in detail. Note that the functional group $-SO_3X$ appears in several places in the tables, where "X" stands for H (sulfonic acid group) or Na (sulfonate salt). In the table we mainly highlight those peaks, which are typical fingerprints for the different terminations.

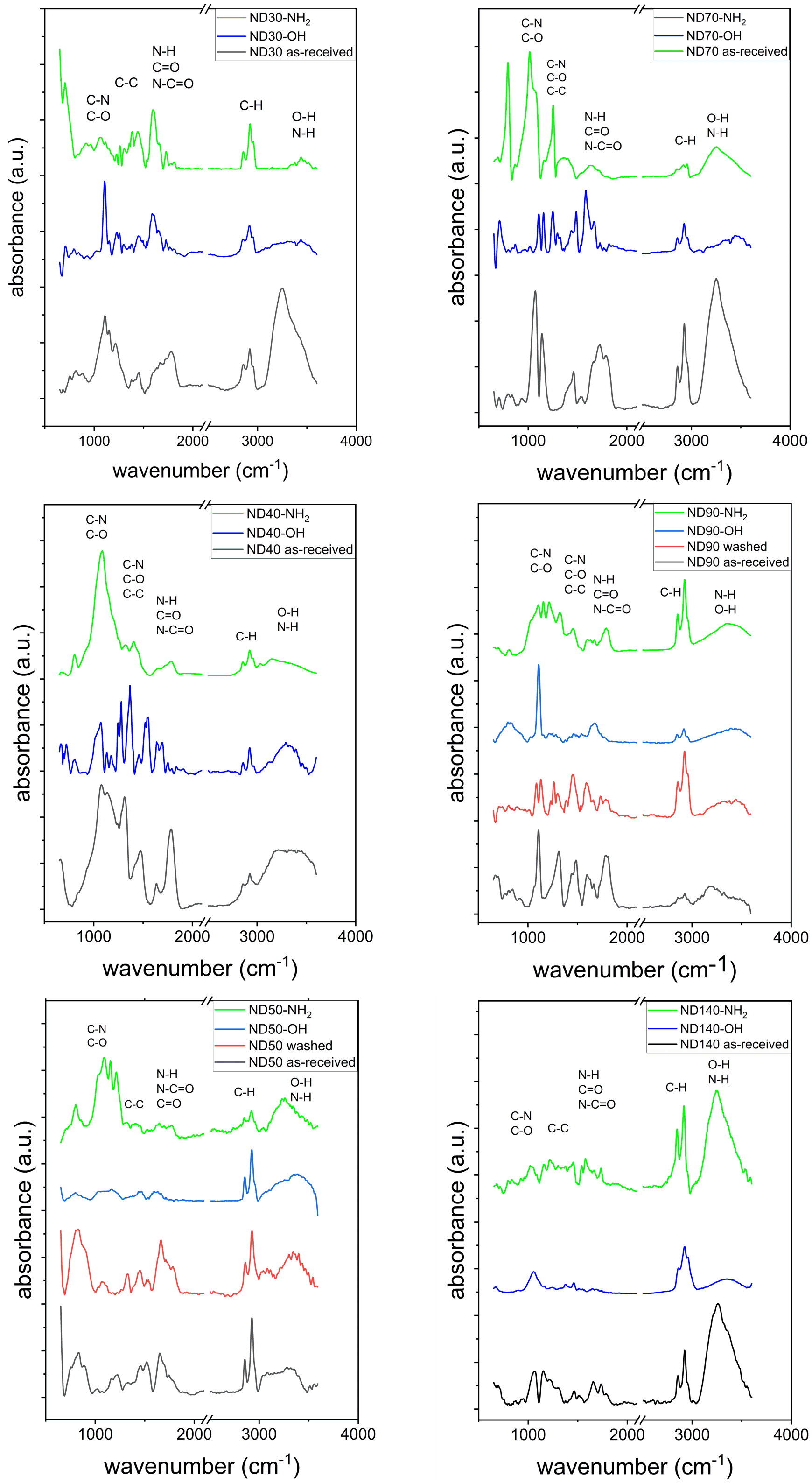

Figure S4: Infrared vibration spectra of the as-received, $-OH$ and $-NH_2$ terminated FNDs with average sizes of 30, 40, 50, 70, 90 and 140 nm

Table S1: FTIR data measured on the ND10 FNDs with various surface terminations. The observed vibration bands, the associated functional groups, and the respective vibration modes are given.

band (cm^{-1})	functional groups	vibration modes
ND10 as-received		
3590–3130	COOH, OH	O–H stretching
2960–2856	CH_3, CH_2	C–H stretching
1850–1660	COOH, CO, CHO, C=C	C=O from carboxyl group (monomer, dimer), acid-anhydride (symmetric and asymmetric), H bond in chelate bond, C=C stretching
1440	CH_3, C–C	bending mode, perpendicular to the plane, C–C skeletal
1270–1180	SO_3X, C–O–C	from sulfonate, symmetric and asymmetric C–O–C stretching
1180–1000	OH	C–O stretching modes (primary, secondary, tertiary)
ND10 −OH terminated		
3600–3120	OH	O–H stretching
2953–2850	CH_3, CH_2	C–H stretching
1660	C=O	C=O from unreacted COOH group, C=C stretching
1463	CH_3	bending mode, perpendicular to the plane
1240–1000	C–O, C–C	C–O stretching modes (primary, secondary, tertiary), skeletal vibration
ND10 $-NH_2$ terminated		
3320	NH_2, NH	N–H stretching
2960–2855	CH_3, CH_2	C–H stretching
1740	CHO, C=O	C=O stretching
1670	CHO	ortho-hydroxy, amino and formyl group, chelate H bond
1650–1540	NH_2, NH	N–H bending
1467	CH_3	bending mode, perpendicular to the plane
1390	C–N	aniline-like and tertiary C–N stretching
1190–1050	C–N	secondary, primary C–N stretching

Table S2: FTIR data measured on the ND30 FNDs with various surface terminations. The observed vibration bands, the associated functional groups, and the respective vibration modes are given.

band (cm^{-1})	functional groups	vibration modes
ND30 as-received		
3550–3090	COOH, OH	O–H stretching
2980–2815	CH_3, CH_2	C–H stretching
1860–1560	CO, CHO, OH	C=O from carboxyl group (monomer, dimer), acid anhydride (symmetric and asymmetric), O–H in intramolecular H-bond, chelate bond
1471	CH_3, C–C	bending mode, perpendicular to the plane, C–C skeletal
1300–1000	SO_3X, C–O–C, C–O	from sulfonate; symmetric and asymmetric C–O–C stretching; C–O stretching (primary, secondary, tertiary)
ND30 −OH terminated		
3500–3120	OH	O–H stretching
2980–2790	CH_3, CH_2	C–H stretching
2100–1930	OH	intramolecular H bond, chelate bond
1820–1700	CHO, CO	unreacted CHO and CO groups, chelate H bond
1680–1550	CHO, CO	C=O stretching; unreacted CHO and O–H in chelate H bond (ortho position), adsorbed water
1450	CH_3	bending mode, perpendicular to the plane
1300–1100	C–O, C–C	C–O stretching modes (primary, secondary, tertiary); skeletal vibration
710	OH	O–H deformation mode in H-bond
ND30 $-NH_2$ terminated		
3550–3300	NH_2, NH	N–H stretching
2980–2825	CH_3, CH_2	C–H stretching
1733	$CONH_2$, CONH	unreacted amide groups (cyclic amide, lactam)
1640–1560	NH_2, NH	N–H bending
1450	CH_3	bending mode, perpendicular to the plane
1390–1050	C–N	aniline-like and tertiary, secondary, primary C–N stretching
705	NH_2	N–H bending perpendicular to the plane

Table S3: FTIR data measured on the ND40 FNDs with various surface terminations. The observed vibration bands, the associated functional groups, and the respective vibration modes are given.

band (cm^{-1})	functional groups	vibration modes
ND40 as-received		
3600–3010	COOH, OH	O–H stretching
2970–2840	CH_3, CH_2	C–H stretching
1860–1620	CO, CHO, OH	C=O from carboxyl group (monomer, dimer), acid anhydride (symmetric and asymmetric), O–H in intramolecular H-bond, chelate bond
1473	CH_3, C–C	bending mode, perpendicular to the plane, C–C skeletal
1350	SO_3X, C–O–C, C–O	from sulfonate; symmetric and asymmetric C–O–C stretching; C–O stretching (primary, secondary, tertiary)
ND40 −OH terminated		
3520–3100	OH	O–H stretching
2980–2790	CH_3, CH_2	C–H stretching
1720–1600	CHO, CO	unreacted CHO and CO groups; chelate H bond
1680–1550	CHO, CO, C=C, OH	C=O stretching; unreacted CHO and O–H in chelate H bond (ortho position); adsorbed water; C=C stretching
1450	CH_3	bending mode, perpendicular to the plane
1360	OH	O–H bending
1290–1220	C–O–C, C–O	C–O stretching (secondary, tertiary)
1080–1000	C–O	C–O stretching (primary, secondary)
720	OH	O–H deformation mode in H-bond
ND40 $-NH_2$ terminated		
3540–3090	NH_2, NH	N–H stretching
2980–2830	CH_3, CH_2	C–H stretching
1845–1580	$CONH_2$, CONH, NH_2, NH	unreacted amide groups (cyclic amide, lactam); N–H bending
1450	CH_3	bending mode, perpendicular to the plane
1410	amide III band	C–N stretching, NH_2 bending, C=O stretching
1320–1000	C–N	aniline-like and tertiary, secondary, primary C–N stretching
806	NH_2	N–H bending perpendicular to the plane

Table S4: FTIR data measured on the ND50 FNDs with various surface terminations. The observed vibration bands, the associated functional groups, and the respective vibration modes are given.

band (cm^{-1})	functional groups	vibration modes
ND50 as-received		
3450–3000	COOH, OH	O–H stretching
2925, 2855	CH_3, CH_2	C–H stretching
1835–1605	COOH, CO, CHO, C=C	C=O from carboxyl group (monomer, dimer), acid anhydride (symmetric and asymmetric), H bond in chelate bond, C=C stretching
1522	C=O, C=C, C–C	skeletal vibrations; C=O from diketone
1270–1130	C–O	tertiary, secondary and aryl-like C–O; asym./sym. C–O–C
1390	C–O	stretching (tertiary)
1190–1050	C–O	stretching (primary, secondary)
890–835	C–O–C	asymmetric/symmetric stretching from dioxane and epoxide-like C–O–C units
ND50 −OH terminated		
3600–3000	OH	O–H stretching
2928, 2855	CH_3, CH_2	C–H stretching
1230–1000	C–O	tertiary, secondary and aryl-like C–O
ND50 $-NH_2$ terminated		
3250	NH_2, NH	N–H associated form
1760–1680	CHO, C=O	ortho-hydroxy, amino and formyl group; chelate H bond
1220–1080	C–N	C–N stretching
800	NH_2	N–H bending mode, perpendicular to the plane

Table S5: FTIR data measured on the ND70 FNDs with various surface terminations. The observed vibration bands, the associated functional groups, and the respective vibration modes are given.

band (cm^{-1})	functional groups	vibration modes
ND70 as-received		
3530–3090	COOH, OH	O–H stretching
2980–2830	CH_3, CH_2	C–H stretching
1980–1800	OH, CO, CHO	C=O from carboxyl group (monomer, dimer), acid anhydride (symmetric and asymmetric), O–H in intramolecular H-bond
1800–1600	CO, CHO, OH, C=C	C=O from carboxyl group (monomer, dimer), acid anhydride (symmetric and asymmetric), O–H in intramolecular H-bond, chelate bond, C=C stretching
1455	CH_3	bending mode, perpendicular to the plane, C–C skeletal
1220–1010	SO_3X, C–O–C, C–O	from sulfonate; symmetric and asymmetric C–O–C stretching; C–O stretching (primary, secondary, tertiary)
ND70 −OH terminated		
3580–3110	OH	O–H stretching
2990–2830	CH_3, CH_2	C–H stretching
1980–1800	OH, CO, CHO	C=O from carboxyl group (monomer, dimer), acid anhydride (symmetric and asymmetric), O–H in intramolecular H-bond
1730–1550	CHO, CO	C=O stretching; unreacted CHO, C=O and O–H in chelate H bond (ortho position); adsorbed water; C=C stretching
1480, 1440	OH, CH_3	bending mode, perpendicular to the plane
1300	OH	O–H bending
1250, 1160, 1100	C–O	C–O stretching (primary, secondary, tertiary)
710	OH	O–H deformation mode in H-bond
ND70 $-NH_2$ terminated		
3540–3070	NH_2, NH	N–H stretching
2975–2800	CH_3, CH_2	ortho-hydroxy, amino and formyl group, chelate H bond
1770–1490	$CONH_2$, CONH, NH_2, NH	unreacted amide groups (cyclic amide, lactam), N–H bending
1460–1300	CH_3, CH_2, C–N	C–H bending mode, perpendicular to the plane; C–N stretching (aniline-like)
1250	C–N	tertiary, secondary C–N stretching
1080–1020	C–N	secondary, primary C–N stretching
795	NH_2	N–H bending perpendicular to the plane

Table S6: FTIR data measured on the ND90 FNDs with various surface terminations. The observed vibration bands, the associated functional groups, and the respective vibration modes are given.

band (cm^{-1})	functional groups	vibration modes
ND90 as-received		
3380–3050	COOH, OH	O–H stretching
1790	C=O	C=O from acid anhydride, symmetric and antisymmetric
1690–1600	C=O	C=O from carboxyl group
1480	CH_3, C–C	bending mode, perpendicular to the plane, C–C skeletal vibrations
1310	C–O, S=O	symmetric C–O stretching (CO–O) and S=O from sulfonate
1110	C–O, C–O–C	C–O stretching from C–OH, anhydride and ether-like
ND90 −OH terminated		
3600–3070	OH	O–H stretching
2960–2845	CH_3, CH_2	could be the C=O of an unreacted COOH group, or chelate bond seen by the ortho OH–COOH group; β-diketo carbonyl moiety
1110	C–O	C–O stretching mostly from secondary alcoholic group
ND90 $-NH_2$ terminated		
3370	NH_2, NH	N–H stretching
2970–2855	CH_3, CH_2	C–H stretching
1785	CHO, C=O	ortho-hydroxy/amino and formyl group, chelate H bond
1650–1560	NH_2, NH	primary and secondary N–H bending mode
1460	CH_3, C–C	bending mode, perpendicular to the plane; C–C skeletal mode
1320	C–N	aromatic-like and secondary C–N stretching
1212–1100	C–N	primary and secondary C–N stretching
808	NH_2	N–H bending mode, perpendicular to the plane

Table S7: FTIR data measured on the ND140 FNDs with various surface terminations. The observed vibration bands, the associated functional groups, and the respective vibration modes are given.

band (cm^{-1})	functional groups	vibration modes
ND140 as-received		
3600–3060	COOH, OH	O–H stretching
2980–2830	CH_3, CH_2	C–H stretching
1820–1620	COOH, CO, CHO	C=O from carboxyl group (monomer, dimer), acid anhydride (symmetric and asymmetric)
1460	CH_3, C–C	bending mode, perpendicular to the plane, C–C skeletal
1350–1120	SO_3X, C–O–C, C–O	from sulfonate; symmetric and asymmetric C–O–C stretching; C–O stretching (secondary, tertiary)
1180–1000	C–O	C–O stretching modes (primary, secondary, tertiary)
ND140 −OH terminated		
3550–3100	OH	O–H stretching
2980–2830	CH_3, CH_2	C–H stretching
1740–1600	CHO, CO	C=O from unreacted carboxyl group (monomer, dimer); CHO formyl group from reduced COOH (C=O symmetric and asymmetric stretching)
1460	CH_3	bending mode, perpendicular to the plane
1150–980	C–O, C–C	C–O stretching modes (primary, secondary, tertiary); skeletal vibration
ND140 $-NH_2$ terminated		
3500–3080	NH_2, NH	N–H stretching
2960–2790	CH_3, CH_2	C–H stretching
1730	C=O	C=O stretching from unreacted amide (lactam, cyclic amide)
1670	CHO, NH	ortho-hydroxy, amino and formyl group, chelate H bond
1580–1540	NH_2, NH	N–H bending
1457	CH_3	bending mode, perpendicular to the plane
1390–1160	C–N	aniline-like and tertiary C–N stretching
1060–1040	C–N	secondary amino C–N stretching

3 XPS supporting data

The XPS spectra of the C and N core levels for the ND50, ND90, and ND140 samples are presented in Fig. S5. Please refer to the main text for the detailed interpretation of the peak assignments.

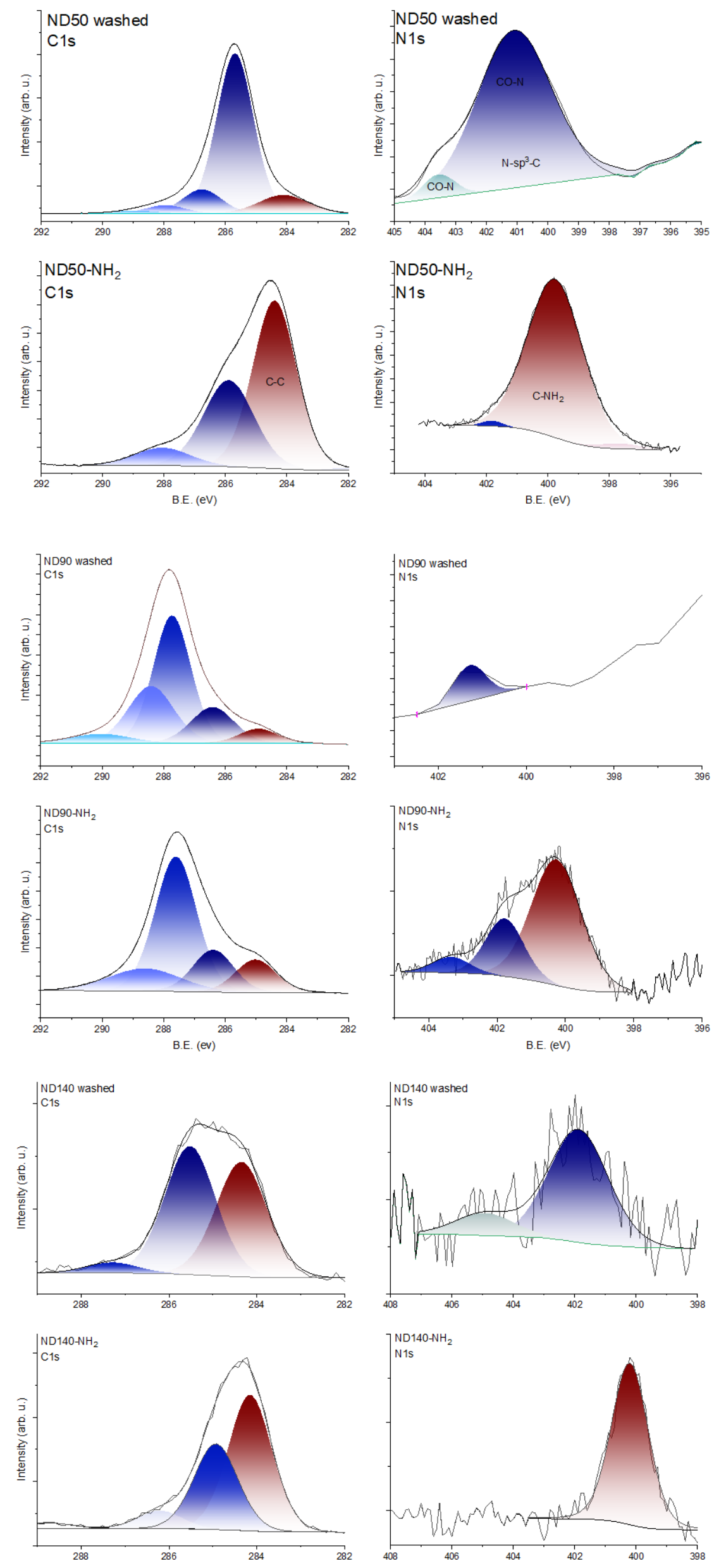

Figure S5: The XPS spectra of C and N elements in ND50, ND90 and ND140 FNDs

4 Additional photoluminescence (PL) data

The NV(−) emission fraction was determined for the as-received FNDs as a function of the applied laser power (Fig. S6). The corresponding results for the washed, −OH-terminated, and $-NH_2$-terminated FNDs, measured over an extended range of laser powers, are presented in Fig. S7.

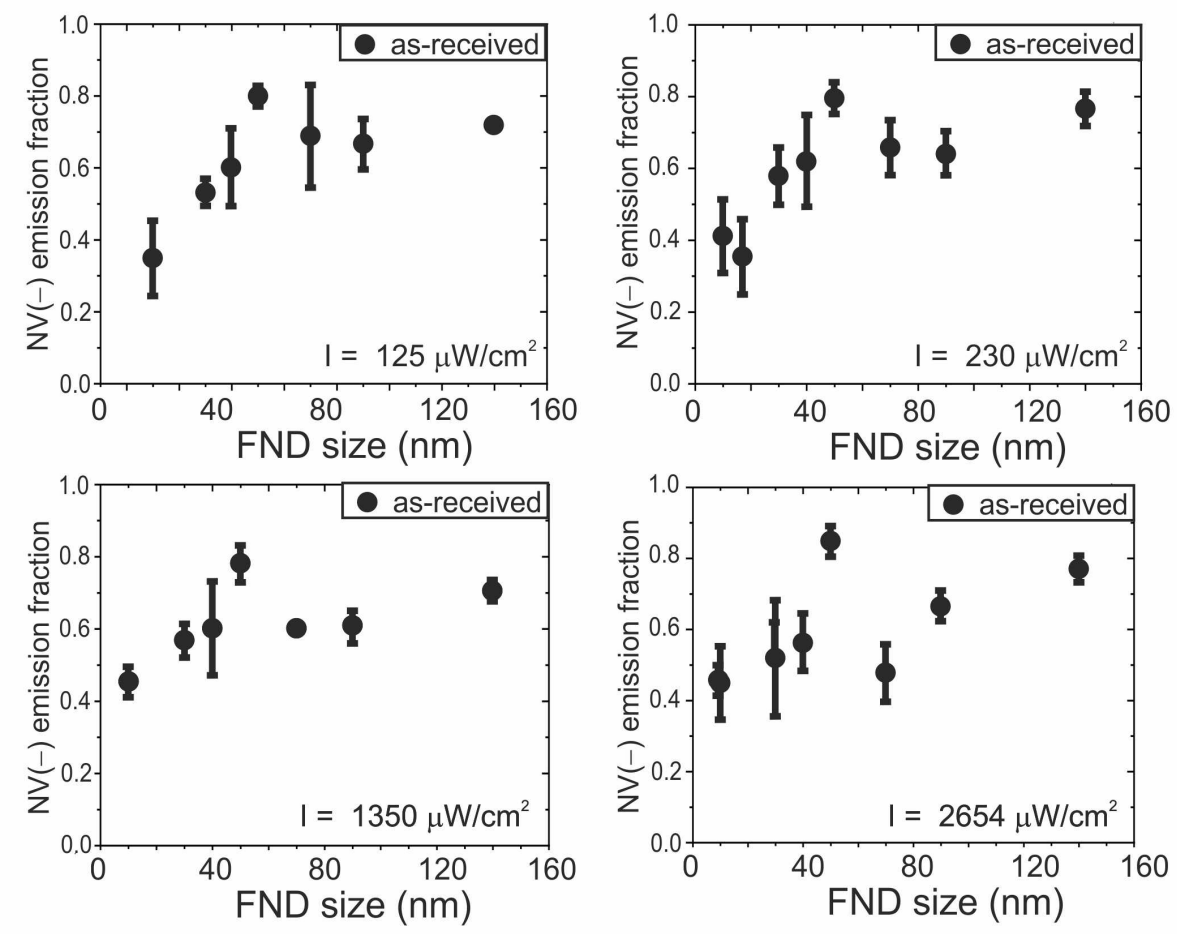

Figure S6: NV(−) emission fraction for the as-received FNDs as a function of the applied laser power for different FND sizes.

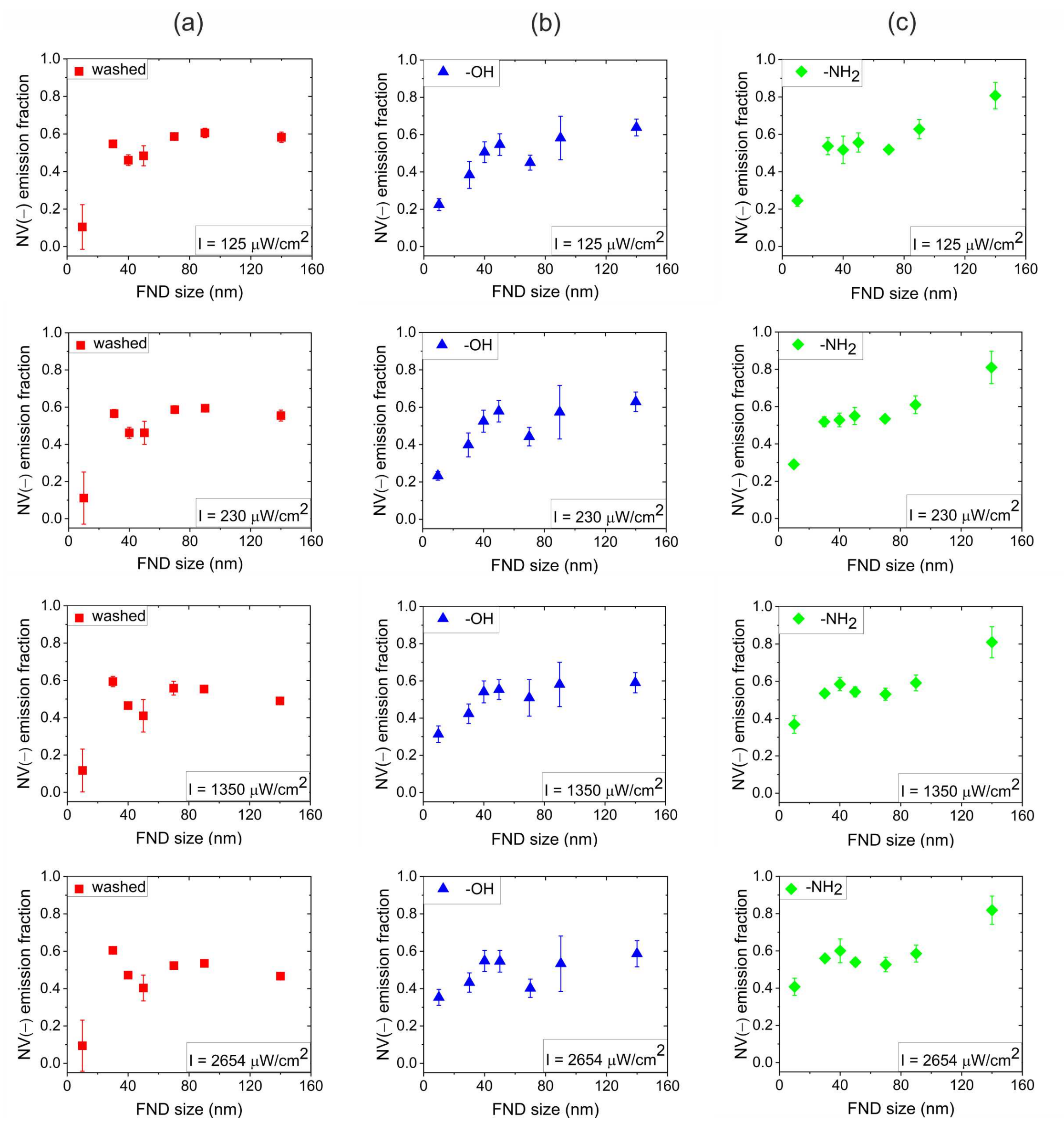

Figure S7: Photoluminescence spectrum analysis of FNDs. NV(−) emission fraction for the (a) washed, (b) −OH terminated, and (c) $-NH_2$ terminated FNDs as a function of the applied laser intensity for different FND sizes.
